# Supplementary material for: Splice-Junction-Based Mapping of Alternative Isoforms in the Human Proteome
Source: Cell Rep. Author manuscript; Available in PMC 2020 Jan 15. (PMC6961840; doi:10.1016/j.celrep.2019.11.026)
Supplement: 2 [file NIHMS1546469-supplement-2.zip › DF1/PXD006675/AtrialSeptum/AtrialSeptum_18_TPM3_AISEELDHALNDMTSG.pdf]

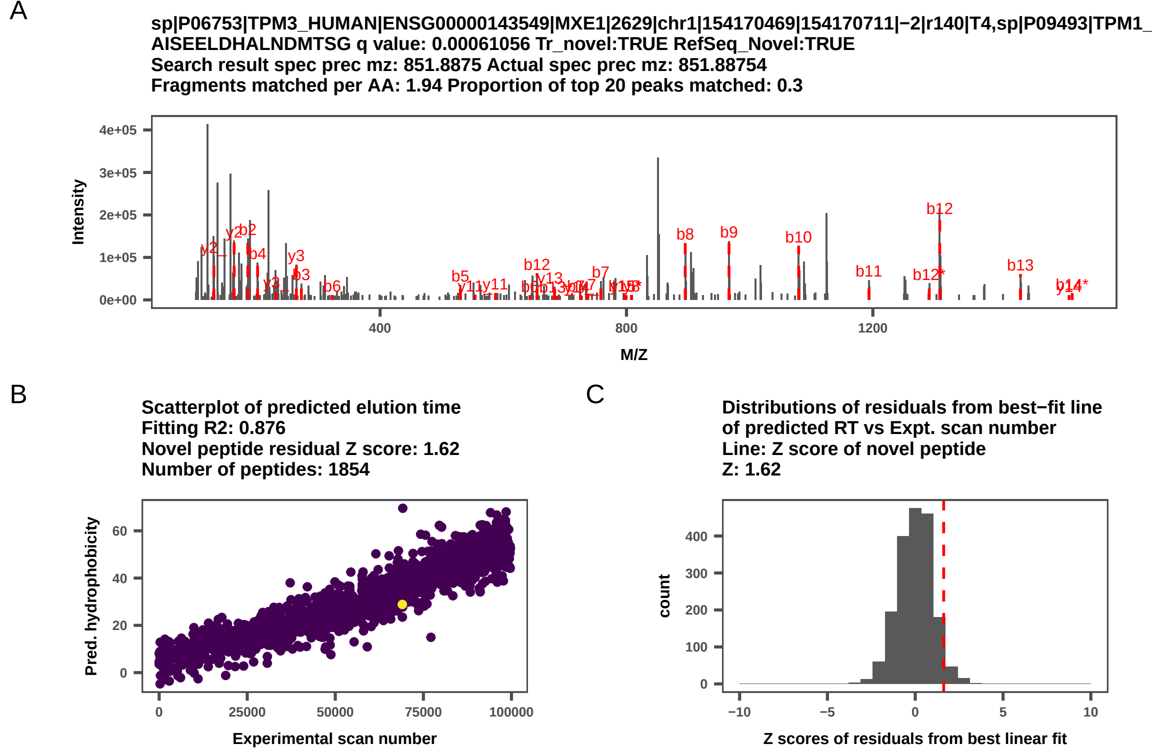

A
sp|P06753|TPM3_HUMAN|ENSG00000143549|MXE1|2629|chr1|154170469|154170711|−2|r140|T4,sp|P09493|TPM1_
AISEELDHALNDMTSG q value: 0.00061056 Tr_novel:TRUE RefSeq_Novel:TRUE
Search result spec prec mz: 851.8875 Actual spec prec mz: 851.88754
Fragments matched per AA: 1.94 Proportion of top 20 peaks matched: 0.3
Intensity
M/Z
B
Scatterplot of predicted elution time
Fitting R2: 0.876
Novel peptide residual Z score: 1.62
Number of peptides: 1854
Pred. hydrophobicity
Experimental scan number
C
Distributions of residuals from best−fit line
of predicted RT vs Expt. scan number
Line: Z score of novel peptide
Z: 1.62
count
Z scores of residuals from best linear fit
